# Supplementary figures and images for: Hip, knee, and ankle joint forces during exoskeletal-assisted walking: Comparison of approaches to simulate human-robot interactions
Source: PLoS One. 2025 Aug 29;20(8):e0322247. doi: 10.1371/journal.pone.0322247 (PMC12396643; doi:10.1371/journal.pone.0322247)

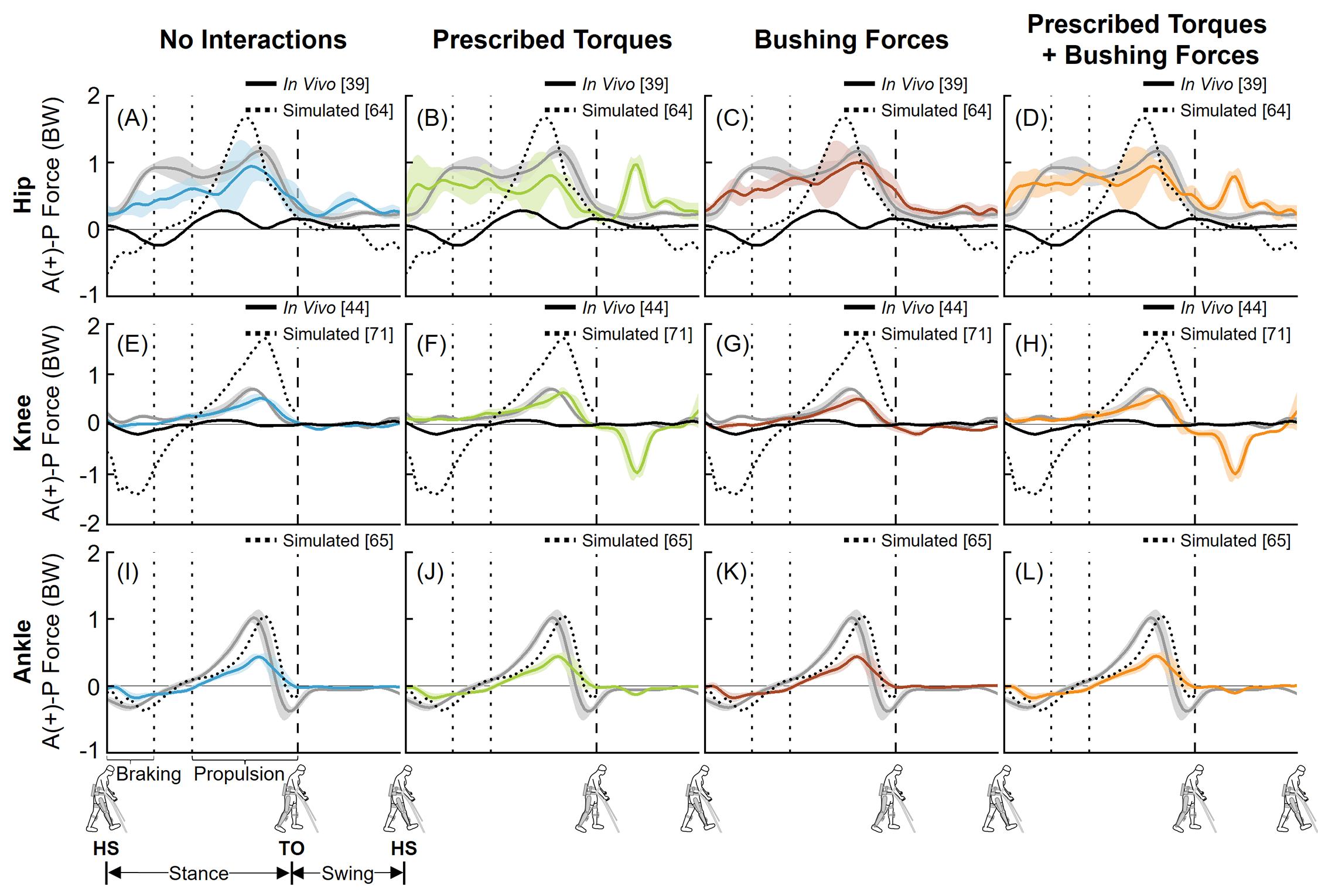

Supplement: S1 Fig — Average (±1 SD) hip (A-D), knee (E-H), and ankle (I-L) joint forces during EAW (six trials, colored lines) were compared to unassisted walking (five trials, grey lines) and previously published in vivo (solid black lines) and simulated (dotted black lines) joint forces during unassisted walking. The joint forces were normalized to the participant’s body weight (BW). Average toe-off from all unassisted walking and EAW trials is represented by dashed vertical lines. The braking and propulsion phases of gait during unassisted walking are represented by dotted vertical lines. (TIF) [file pone.0322247.s001.tif]

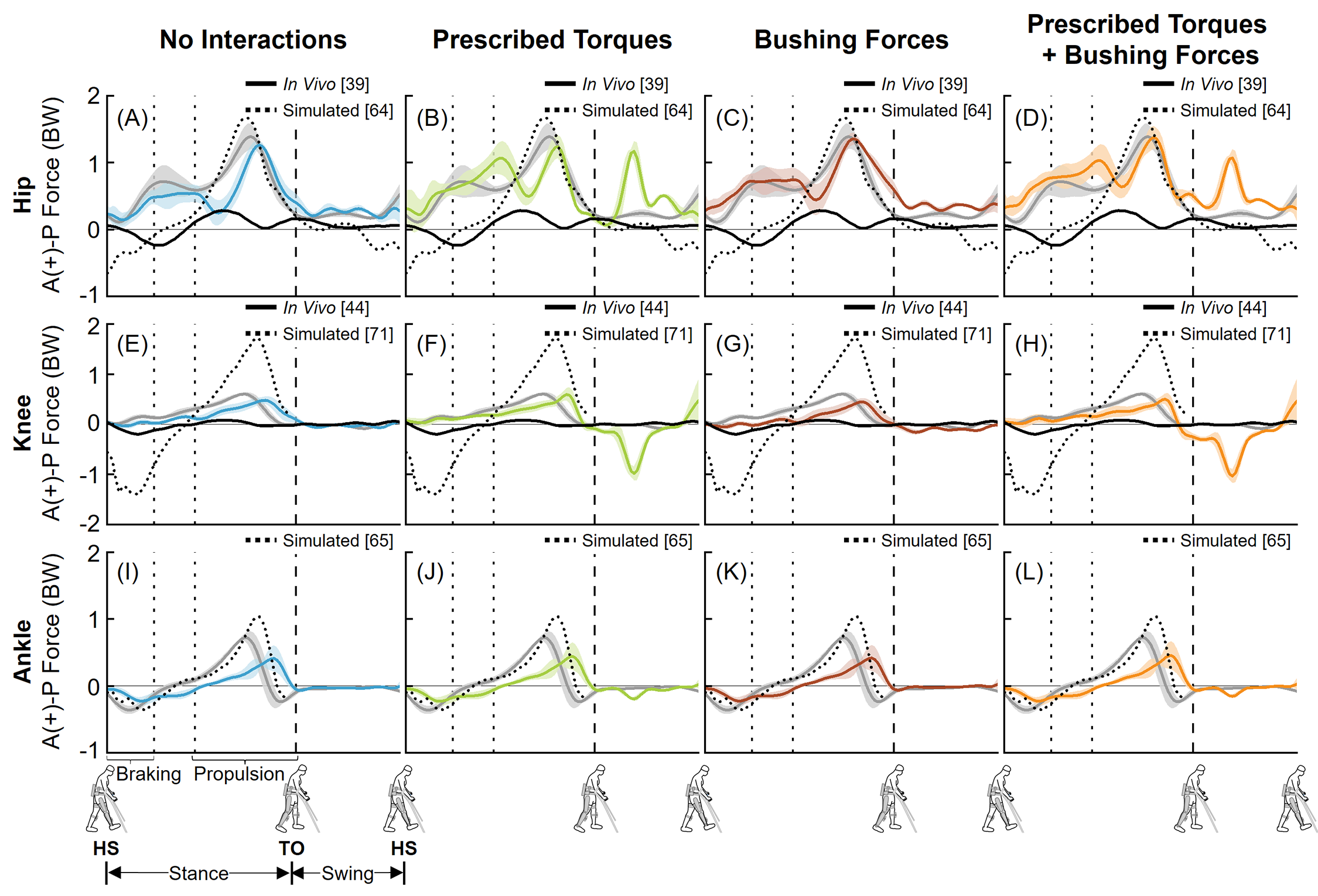

Supplement: S2 Fig — Average (±1 SD) hip (A-D), knee (E-H), and ankle (I-L) joint forces during EAW (six trials, colored lines) were compared to unassisted walking (five trials, grey lines) and previously published in vivo (solid black lines) and simulated (dotted black lines) joint forces during unassisted walking. The joint forces were normalized to the participant’s body weight (BW). Average toe-off from all unassisted walking and EAW trials is represented by dashed vertical lines. The braking and propulsion phases of gait during unassisted walking are represented by dotted vertical lines. (TIF) [file pone.0322247.s002.tif]

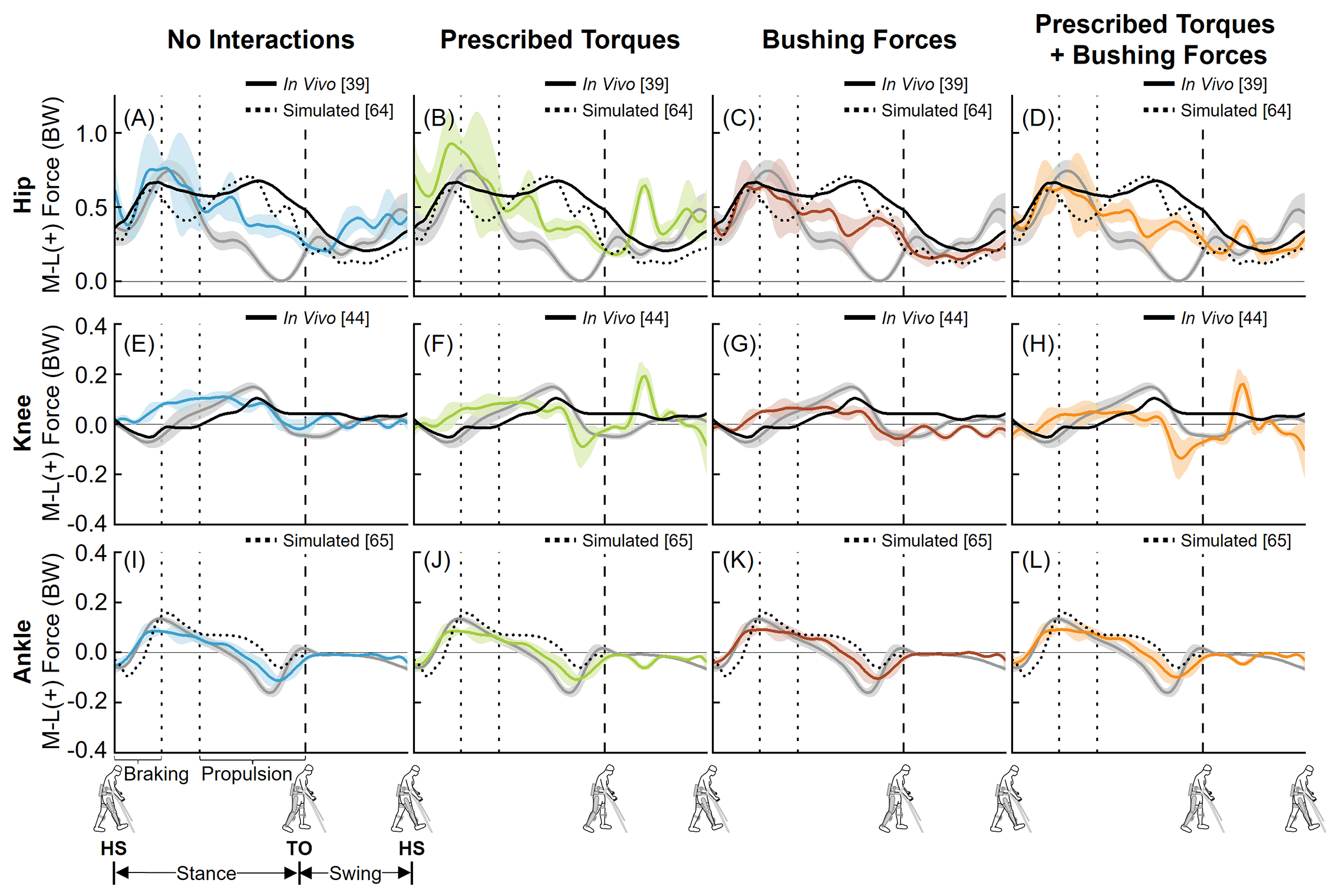

Supplement: S3 Fig — Average (±1 SD) hip (A-D), knee (E-H), and ankle (I-L) joint forces during EAW (six trials, colored lines) were compared to unassisted walking (five trials, grey lines) and previously published in vivo (solid black lines) and simulated (dotted black lines) joint forces during unassisted walking. The joint forces were normalized to the participant’s body weight (BW). Average toe-off from all unassisted walking and EAW trials is represented by dashed vertical lines. The braking and propulsion phases of gait during unassisted walking are represented by dotted vertical lines. (TIF) [file pone.0322247.s003.tif]

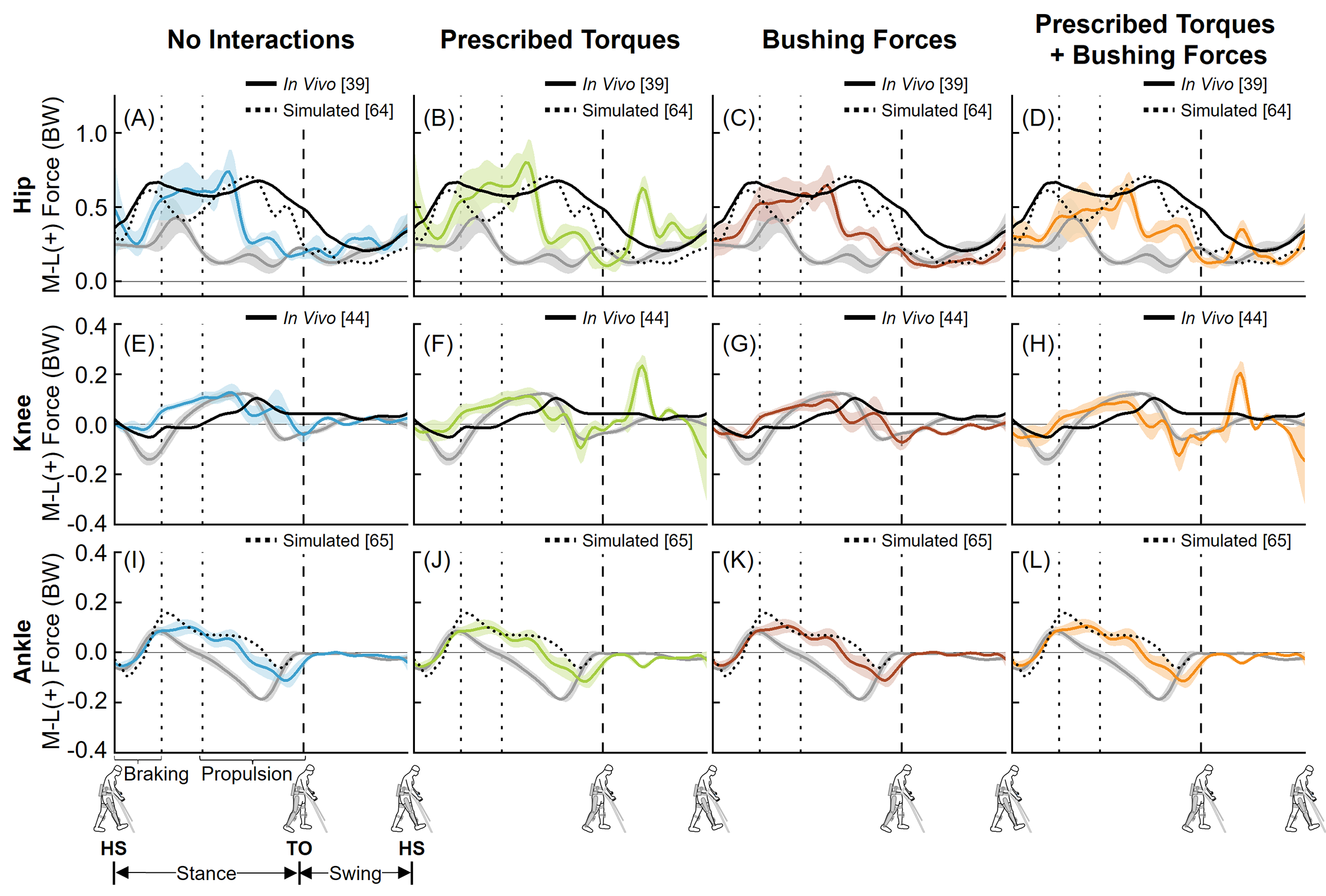

Supplement: S4 Fig — Average (±1 SD) hip (A-D), knee (E-H), and ankle (I-L) joint forces during EAW (six trials, colored lines) were compared to unassisted walking (five trials, grey lines) and previously published in vivo (solid black lines) and simulated (dotted black lines) joint forces during unassisted walking. The joint forces were normalized to the participant’s body weight (BW). Average toe-off from all unassisted walking and EAW trials is represented by dashed vertical lines. The braking and propulsion phases of gait during unassisted walking are represented by dotted vertical lines. (TIF) [file pone.0322247.s004.tif]

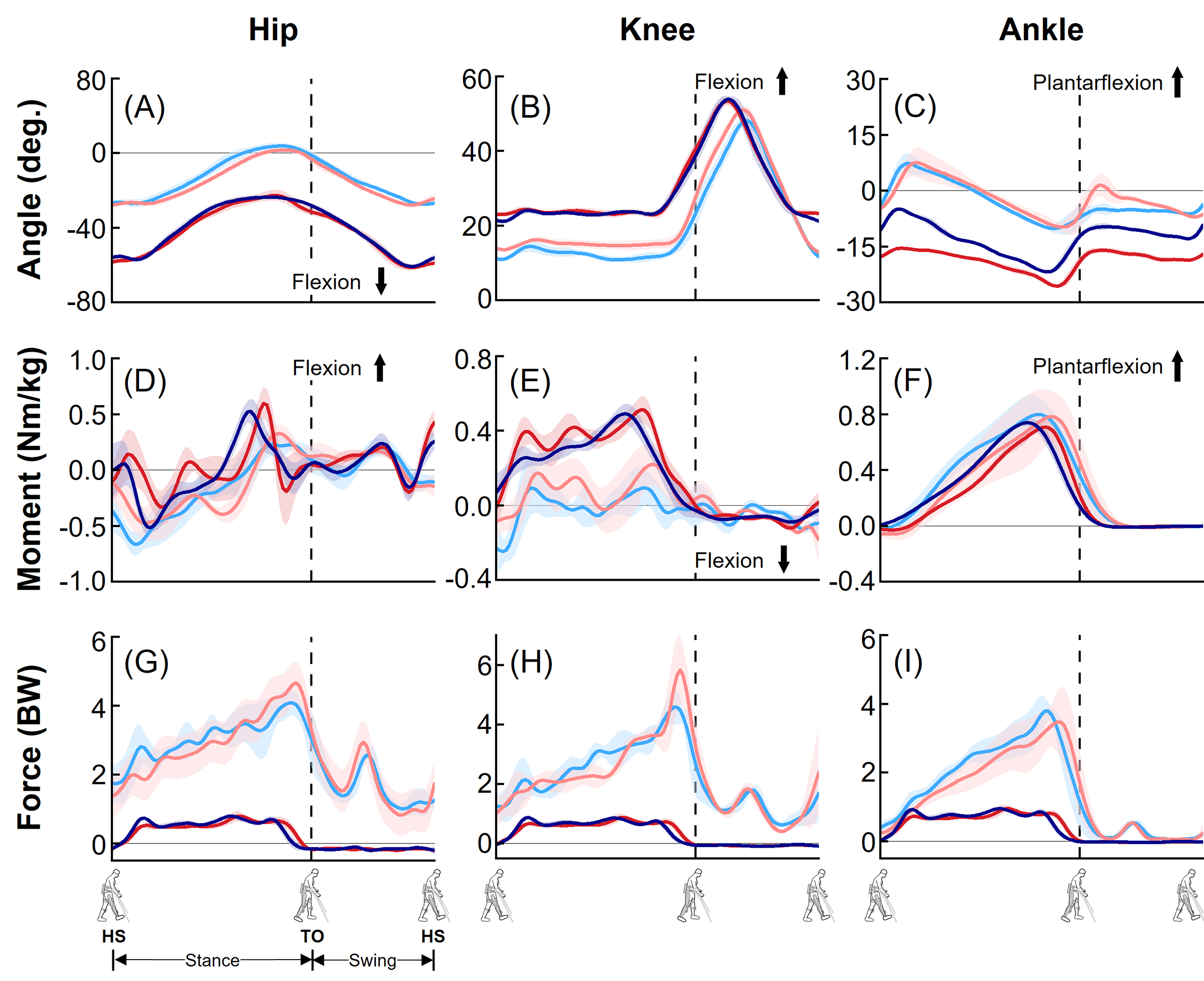

Supplement: S5 Fig — The joint moments were normalized to the combined mass of each participant and the exoskeleton. The joint forces were normalized to each participant’s body weight (BW). Average toe-off from all EAW trials of the participant with SCI is represented by dashed vertical lines. (TIF) [file pone.0322247.s005.tif]
