# Supplementary material for: Hip, knee, and ankle joint forces during exoskeletal-assisted walking: Comparison of approaches to simulate human-robot interactions
Source: PLoS One. 2025 Aug 29;20(8):e0322247. doi: 10.1371/journal.pone.0322247 (PMC12396643; doi:10.1371/journal.pone.0322247)
Supplement: Attachment — Submitted filename: Comments.pdf [file pone.0322247.s006.pdf]

This manuscript presents a computational framework for estimating hip, knee, and ankle joint forces during exoskeletal-assisted walking (EAW) using the ReWalk P6.0 device. The study compares four simulation approaches for modeling human-robot interaction and validates the framework by comparing simulated results with in vivo and prior simulation data. The manuscript is methodologically thorough and well-written, with detailed justification for modeling decisions and simulation parameters. However, despite its progress, significant concerns remain unaddressed. Some of the raised concerns are mentioedn below:

Major Concerns

1. **Single-Participant Limitation**
   - While the authors justify the use of an able-bodied participant for validation, the generalizability of the findings is extremely limited. The target population (SCI patients) differs substantially in musculoskeletal structure, motor control, and muscle tone, and this should be better contextualized. A plan to extend the framework to SCI users is only briefly mentioned and should be strengthened or supplemented with pilot data.
2. **Absence of Crutch Force Data**
   - The omission of external crutch forces in the simulation is a major limitation, as these are likely to offload lower limb joints significantly. Their exclusion could overestimate joint loading during EAW. This should be addressed explicitly in both the limitations and in the interpretation of results.
3. **No Ground Truth for EAW**
   - There is no in vivo or experimental reference for joint loads during EAW, making it difficult to assess the validity of the computed forces. While the comparison to unassisted walking and other simulations is useful, the manuscript should make clearer that absolute accuracy of EAW predictions remains uncertain.
4. **Statistical Analysis Lacking**
   - While the authors provide average and standard deviation values and RMS errors, there is a lack of formal statistical comparison (e.g., ANOVA or t-tests) between simulation conditions or against prior studies. Even though n=1, formal statistical testing across trials (where possible) would add rigor.
5. **Figures and Interpretability**
   - Figures are numerous and detailed but may be overwhelming to readers. Consider consolidating or simplifying where appropriate. Moreover, the use of color coding across simulation methods should be standardized across all plots to aid interpretation.

Minor Concerns

- **Terminology**: The term "functionally equivalent to physical EAW" may be too strong when describing the "Prescribed Torques + Bushing Forces" method. Consider tempering this language.

- **Rationale for EMG Muscles**: A clearer rationale for selecting the eight muscles used for EMG tracking would improve transparency.
- **Clarify Impact of Torques vs. Bushing Forces**: While the distinction between methods is clear, a clearer discussion of their individual biomechanical impact on each joint would improve mechanistic interpretation.
- **Literature Citations**: Some references, such as those for previously published in vivo data, are briefly mentioned but not thoroughly discussed. More critical evaluation of how current findings extend or deviate from prior work is needed.

Recommendations for Improvement

1. Consider conducting simulations on data from at least one SCI participant, even if exploratory, to illustrate feasibility and differences.
2. Quantify the potential error introduced by the exclusion of crutch forces—either via sensitivity analysis or literature estimates.
3. Improve clarity in figures, possibly through simplification or use of summary plots comparing peak forces across methods.
4. Include formal statistical tests, even within-subject, where appropriate.
5. References: The following refeercnes are highly recommended toa dded to the paper to improve its quality and state of the arts. (10.1007/s41315-025-00421-x), (10.22024/UniKent/01.02.105160), and (10.1109/ACCESS.2023.3325211).
