# Supplementary material for: Hip, knee, and ankle joint forces during exoskeletal-assisted walking: Comparison of approaches to simulate human-robot interactions
Source: PLoS One. 2025 Aug 29;20(8):e0322247. doi: 10.1371/journal.pone.0322247 (PMC12396643; doi:10.1371/journal.pone.0322247)
Supplement: Attachment — Submitted filename: Review questions.pdf [file pone.0322247.s007.pdf]

The manuscript entitled ‘’Hip, knee, and ankle joint forces during exoskeletal-assisted walking: comparison of approaches to simulate human-robot interactions” has been organized and developed in good shape. The overall goal of this study was to develop a computational framework to quantify hip, knee, and ankle joint forces during exoskeletal-assisted walking (EAW) in the ReWalk P6.0, an FDA approved lower-extremity exoskeleton. It has 2 main objectives: The first objective was to quantify hip, knee, and ankle joint forces during unassisted walking and compare the results to existing *in vivo* and simulation data. The second objective was to compare hip, knee, and ankle joint forces from four different approaches to simulate human-robot interactions during EAW. OpenSim Moco is used to determine the joint reaction forces at the hips, knees, and ankles during unassisted walking and EAW. The study is well-developed and the results are intriguing. Once the following comments are addressed, the manuscript is recommended for publication.

Here are several **technical review questions** that need to be answered.

1. Page 6 line 133: What are the training protocols for the experiment? Describe here or at least provide it in supplementary document?
2. Page 7 line 142: What is the experiment procedure, describe briefly?
3. Page 8 line 180: What are the other cost terms here? Why you are using Opensim MOCO, as static optimization or CMC also have the same cost function of minimizing the muscle activation? Describe your cost function properly?
4. Page 9 line 194: It is not clear how joint reaction force is quantified?
5. Page 10: What assumptions and parameter were made in Modeling the bushing forces and prescribed torques applied by the exoskeleton?
6. Page 10: How were interaction forces (in the bushing model) distributed across body segments, and how sensitive are the results to these assumptions?
7. Page 12 line 250-255: It is redundant? Justify here.
8. Legend of figure should be clearer. As you explain in figure title at least mention all 3 lines in legend of figure 2.
9. Page 22 line 482: Why joint force at hip and knee for assisted and unassisted is same?
