# Supplementary material for: Hip, knee, and ankle joint forces during exoskeletal-assisted walking: Comparison of approaches to simulate human-robot interactions
Source: PLoS One. 2025 Aug 29;20(8):e0322247. doi: 10.1371/journal.pone.0322247 (PMC12396643; doi:10.1371/journal.pone.0322247)
Supplement: Attachment — Submitted filename: Response to Reviewers.docx [file pone.0322247.s009.docx]

**Response to Reviewers**

We thank the Editor and the Reviewers for their thorough review of our work. We have endeavored to address all critiques. As a result, the quality of this resubmission has been greatly enhanced. The specific responses to the comments are described below, and the changes are highlighted in the revised manuscript. Please note that the sentences in italics are reviewer comments.

**Journal Requirements:**

*1.Please ensure that your manuscript meets PLOS ONE's style requirements, including those for file naming. The PLOS ONE style templates can be found at*

[*https://journals.plos.org/plosone/s/file?id=wjVg/PLOSOne_formatting_sample_main_body.pdf*](https://urldefense.com/v3/__https:/journals.plos.org/plosone/s/file?id=wjVg*PLOSOne_formatting_sample_main_body.pdf__;Lw!!DLa72PTfQgg!Mk5CCMjvFqRFufwB-TdsD2X1CD6hmnDHc9pjr14uP6wa6sGLgQkjAp5XmdvCdMGrHZ4D39DGI0cuwQ$)

*and*

[*https://journals.plos.org/plosone/s/file?id=ba62/PLOSOne_formatting_sample_title_authors_affiliations.pdf*](https://urldefense.com/v3/__https:/track.editorialmanager.com/CL0/https:*2F*2Fjournals.plos.org*2Fplosone*2Fs*2Ffile*3Fid=ba62*2FPLOSOne_formatting_sample_title_authors_affiliations.pdf/1/010f01975839c7ae-270a6174-d3ed-4e7d-823f-aeaf404564a8-000000/h4jbDRq2YUCQhlz_bcgF2NzO-nzWS76G-crckfrE8S8=214__;JSUlJSUlJQ!!DLa72PTfQgg!Mk5CCMjvFqRFufwB-TdsD2X1CD6hmnDHc9pjr14uP6wa6sGLgQkjAp5XmdvCdMGrHZ4D39BZeo0OVw$)

Authors’ Response: We have ensured that the manuscript complies with the style requirements for PLOS ONE.

*2. When completing the data availability statement of the submission form, you indicated that you will make your data available on acceptance. We strongly recommend all authors decide on a data sharing plan before acceptance, as the process can be lengthy and hold up publication timelines. Please note that, though access restrictions are acceptable now, your entire data will need to be made freely accessible if your manuscript is accepted for publication. This policy applies to all data except where public deposition would breach compliance with the protocol approved by your research ethics board. If you are unable to adhere to our open data policy, please kindly revise your statement to explain your reasoning and we will seek the editor's input on an exemption. Please be assured that, once you have provided your new statement, the assessment of your exemption will not hold up the peer review process.*

Authors’ Response: We have a data sharing plan in place. We will share our data through the Figshare database.

*3. Please include your full ethics statement in the ‘Methods’ section of your manuscript file. In your statement, please include the full name of the IRB or ethics committee who approved or waived your study, as well as whether or not you obtained informed written or verbal consent. If consent was waived for your study, please include this information in your statement as well.*

Authors’ Response: The full ethics statement is now included in the Methods section.

*4. Please note that PLOS ONE has specific guidelines on code sharing for submissions in which author-generated code underpins the findings in the manuscript. In these cases, we expect all author-generated code to be made available without restrictions upon publication of the work. Please review our guidelines at* [*https://journals.plos.org/plosone/s/materials-and-software-sharing#loc-sharing-code*](https://urldefense.com/v3/__https:/journals.plos.org/plosone/s/materials-and-software-sharing*loc-sharing-code__;Iw!!DLa72PTfQgg!Mk5CCMjvFqRFufwB-TdsD2X1CD6hmnDHc9pjr14uP6wa6sGLgQkjAp5XmdvCdMGrHZ4D39B_8z2FXg$) *and ensure that your code is shared in a way that follows best practice and facilitates reproducibility and reuse.*

Authors’ Response: We will share our data and models per the best practices.

*Additional Editor Comments:*

*The manuscript presents a computational framework to estimate hip, knee, and ankle joint forces during exoskeleton-assisted walking using the ReWalk P6.0 device, comparing four simulation approaches and validating results against in vivo and prior simulation data. Reviewers acknowledged the methodological rigor and writing quality but highlighted key concerns: limited generalizability due to reliance on a single able-bodied participant, exclusion of crutch forces, lack of ground truth for EAW validation, insufficient statistical analysis, and limited participants. Additional critiques emphasized missing demographic details, unclear method rationale, inadequate result interpretation, and the need for more relevant citations and figures to strengthen the manuscript’s impact and applicability.*

Authors’ Response: We have addressed each of these comments in our revised manuscript.

**Reviewers’ Comments:**

**Reviewer #1**

*This manuscript presents a computational framework for estimating hip, knee, and ankle joint forces during exoskeletal-assisted walking (EAW) using the ReWalk P6.0 device. The study compares four simulation approaches for modeling human-robot interaction and validates the framework by comparing simulated results with in vivo and prior simulation data. The manuscript is methodologically thorough and well-written, with detailed justification for modeling decisions and simulation parameters. However, despite its progress, significant concerns remain unaddressed. Some of the raised concerns are mentioned below:*

*Major Concerns*

1. *Single-Participant Limitation*

- *While the authors justify the use of an able-bodied participant for validation, the generalizability of the findings is extremely limited. The target population (SCI patients) differs substantially in* *musculoskeletal structure, motor control, and muscle tone, and this should be better contextualized. A plan to extend the framework to SCI users is only briefly mentioned and should be strengthened or supplemented with pilot data.*

Authors’ Response: We agree that the generalizability of our joint force results from an able-bodied (AB) participant to persons with SCI is limited. We have added the Reviewer’s concerns in our limitations. We respectfully emphasize that this is a methods paper to describe a novel computational framework. We simulated EAW using four different methods of increasing complexity to gain confidence in our computational framework. Nonetheless, the methodology to compute the intermediate model outputs, including joint kinematics and joint moments, are the same between AB and SCI participants. Per the Reviewer’s request, we have added a supplemental figure (S5 Fig) as pilot data from a participant with SCI during EAW.

2. *Absence of Crutch Force Data*

- *The omission of external crutch forces in the simulation is a major limitation, as these are likely to offload lower limb joints significantly. Their exclusion could overestimate joint loading during EAW. This should be addressed explicitly in both the limitations and in the interpretation of results.*

Authors’ Response: Per the Reviewer’s request, we noted the absence of crutch forces as the second limitation of our study and in the interpretation of results (at the end of third paragraph of Results). We have clarified that the absence of crutch forces likely resulted in overestimation of joint loading during EAW.

3. *No Ground Truth for EAW*

- *There is no in vivo or experimental reference for joint loads during EAW, making it difficult to assess the validity of the computed forces. While the comparison to unassisted walking and other simulations is useful, the manuscript should make clearer that absolute accuracy of EAW predictions remains uncertain.*

Authors’ Response: We have added the Reviewer’s comments as the third limitation of this study.

4. *Statistical Analysis Lacking*

- *While the authors provide average and standard deviation values and RMS errors, there is a lack of formal statistical comparison (e.g., ANOVA or t-tests) between simulation conditions or against prior studies. Even though n=1, formal statistical testing across trials (where possible) would add rigor.*

Authors’ Response: We agree that appropriate statistical methods add scientific rigor and help with data interpretation. Our study includes a single able-bodied participant. From this one participant, we recorded multiple trials of unassisted walking and EAW, which we presented with appropriate average and standard deviation results. Comparison of unassisted walking vs. EAW through statistical tests such as t-tests or ANOVA was not a research objective of this study. Next, we developed virtual simulators of unassisted walking and compared our computed joint forces to prior *in vivo* and simulation studies. Here, we appropriately reported RMS errors; additional comparisons through t-tests or ANOVA with missing data from prior published studies is not statistically sound. Finally, we developed virtual simulators from four different approaches to simulate human-robot interactions during EAW to test the robustness of our computational framework. Comparison of the results from the four different simulation conditions through statistical tests such as t-tests or ANOVA was not a research objective of this study. We have taken the following steps to address this comment: 1) we added a discussion at the end of the “Data analysis and statistical methods” section, and 2) we clarified throughout the manuscript that our second objective was to compute hip, knee, and ankle joint forces from four different approaches to simulate human-robot interactions during EAW in the ReWalk device.

5. *Figures and Interpretability*

- *Figures are numerous and detailed but may be overwhelming to readers. Consider consolidating or simplifying where appropriate. Moreover, the use of color coding across simulation methods should be standardized across all plots to aid interpretation.*

Authors’ Response: The complexity of our study demands detailed figures. We have only included figures that are essential to address the research objectives. Non-essential figures are included as supplementary data. We have described the figures in sufficient detail so the scientific community may reproduce our work. The color coding across simulation methods was standardized across all relevant plots.

*Minor Concerns*

- *Terminology: The term “functionally equivalent to physical EAW” may be too strong when describing the “Prescribed Torques + Bushing Forces” method. Consider tempering this language.*

Authors’ Response: We changed the term to “functionally the most similar to physical EAW”.

- *Rationale for EMG Muscles: A clearer rationale for selecting the eight muscles used for EMG tracking would improve transparency.*

Authors’ Response: We have clarified that these eight muscles are the large lower extremity muscles accessible through surface EMG.

- *Clarify Impact of Torques vs. Bushing Forces: While the distinction between methods is clear, a clearer discussion of their individual biomechanical impact on each joint would improve mechanistic interpretation.*

Authors’ Response: We have highlighted in green throughout the manuscript the individual biomechanical impact of Prescribed Torques vs Bushing Forces.

- *Literature Citations: Some references, such as those for previously published in vivo data, are briefly mentioned but not thoroughly discussed. More critical evaluation of how current findings extend or deviate from prior work is needed.*

Authors’ Response: We present the previously published *in vivo* studies in Introduction to provide motivation for our work. Thorough discussion of these prior studies is beyond the scope of this manuscript. Next, we provided a detailed comparison of our results to prior *in vivo* and simulation studies in the second paragraph of Results. In the second paragraph of Discussion, we highlighted how our joint force results build on existing simulation studies with non-FDA-approved devices. In the third paragraph of Discussion, we highlighted how our joint force results corroborated or deviated from prior work.

*Recommendations for Improvement*

1. *Consider conducting simulations on data from at least one SCI participant, even if exploratory, to illustrate feasibility and differences.*

Authors’ Response: We added results from a participant with SCI as a supplemental figure.

2. *Quantify the potential error introduced by the exclusion of crutch forces—either via sensitivity analysis or literature estimates.*

Authors’ Response: It is difficult to design a meaningful sensitivity analysis without quantifying which portions of EAW gait are affected by crutches and the amount of potential offloading as a percentage of body weight. Prior literature estimates are limited to shoulder joint forces. Two prior studies have modeled crutch-assisted EAW with persons with SCI in a non-FDA-approved exoskeleton to quantify shoulder joint forces (Fournier et al. 2018; Smith et al. 2020). Other experimental and modeling studies have quantified the effects of crutches on non-EAW gait (Haubert et al. 2006; Segura and Piazza 2007; Perez-Rizo et al. 2017). Haubert and colleagues quantified shoulder joint forces during ambulation in persons with SCI using instrumented bilateral forearm crutches (Haubert, Gutierrez et al. 2006). Segura and Piazza investigated the mechanics of ambulation with standard and spring-loaded crutches in able-bodied participants (Segura and Piazza 2007). Perez-Rizo and colleagues developed a biomechanical model to study shoulder biomechanics in persons with SCI walking with crutches in two different gait patterns (Perez-Rizo, Trincado-Alonso et al. 2017). To the best of our knowledge, no prior study has estimated the effects of crutches on lower extremity joint forces. We have added this discussion in the limitations.

3. *Improve clarity in figures, possibly through simplification or use of summary plots comparing peak forces across methods.*

Authors’ Response: We have explained our rationale for the figures earlier. Although summary plots comparing peak forces across methods is a sound suggestion, this would mask many of the interesting differences between the methods. For example, the summary plots would mask the increased hip and knee joint compressive forces during the swing phase with addition of motor torques (Figures 4 and 5), as discussed in the fourth paragraph of Discussion.

4. *Include formal statistical tests, even within-subject, where appropriate.*

Authors’ Response: We have addressed this comment earlier.

*5. References: The following references are highly recommended to add to the paper to improve its quality and state of the arts. (10.1007/s41315-025-00421-x), (10.22024/UniKent/01.02. 105160), and (10.1109/ACCESS.2023.3325211).*

Authors’ Response: We added (10.1007/s41315-025-00421-x) and (10.1109/ACCESS.2023. 3325211). We have not included the PhD dissertation as it is not peer-reviewed.

**Reviewer #2**

*The paper presents the comparative study on Hip, knee, and ankle joint forces during exoskeleton-assisted walking. The work is interesting. The following are the suggestions to incorporate:*

1. *The novelty and outcome of the work are to be re-phrased.*

Authors’ Response: We have clarified the novel contributions of this work throughout the manuscript.

2. *The future use of this work should be clearly explained in order to support the applicability of the outcome of this work.*

Authors’ Response: We have clarified future work in the last paragraph of Discussion.

3. *In the Introduction, more recent relevant work should be cited related to problem formulation.*

Authors’ Response: We added several references in the second and third paragraphs of Introduction. In addition, we added a recent publication on knee loading during EAW in the fourth paragraph of Introduction.

4. *Caption of Fig 1 (A) and (B) is not clear.*

Authors’ Response: We modified the caption to improve clarity.

5. *The authors have not discussed about the participants demographic information such as age, height.*

Authors’ Response: We had previously removed specific identifying information from the manuscript based on the guidelines of medRxiv. Based on the Reviewer’s request, we have added this information back in our manuscript.

6. *How many trials of walking have been used for this study?*

Authors’ Response: We highlighted this information in the second paragraph of “Motion capture experiments during unassisted walking and EAW” section. We collected 10 trials each of unassisted and exoskeletal-assisted walking, but only successful trials were included for further analysis. We have clarified in the Methods section the criteria for a successful trial for each maneuver. Based on these criteria, we obtained five successful trials of unassisted walking and six successful trials of EAW.

7. *Did the virtual simulator generated use the same anthropomorphic dimension as that of each participants?*

Authors’ Response: Yes, we have highlighted this information in the first paragraph of “Virtual simulator reproducing unassisted walking and EAW” section.

8. *The presentation of study is not good. The multiple things cause confusion to the reader. Please manage the subsections of methods section appropriately.*

Authors’ Response: We concur with the Reviewer that the presentation format is somewhat confusing. The appearance of the figure captions directly after the paragraph in which they are first cited, which is a requirement for this journal, takes away from the clarity of the text. Furthermore, we organized the subsections per the guidelines of this journal. We are confident that these issues will be resolved in the proof stage of the manuscript.

9. *The description of Results is not sufficient and can be improved further.*

Authors’ Response: We have clearly described the results to support our research objectives.

10. *Why did authors choose those four specific methods for EAW.*

Authors’ Response: This is a good question. The four different approaches provide a relatively complete representation of the dynamics of EAW. We have added this sentence in the last paragraph of Introduction.

11. *References are not justified*

Authors’ Response: We have changed the formatting of the references per the Reviewer’s request.

12. *Discussion can be re-phrased in order to enhance clarity of the study.*

Authors’ Response: We have made many changes to the Discussion section to improve clarity.

13. *The study should be extended for experiments with SCI participants.*

Authors’ Response: Yes, we are actively working on expanding this study framework to persons with SCI. We have added a supplemental figure as pilot data from a participant with SCI.

**Reviewer #3**

*The manuscript entitled “Hip, knee, and ankle joint forces during exoskeletal-assisted walking: comparison of approaches to simulate human-robot interactions” has been organized and developed in good shape. The overall goal of this study was to develop a computational framework to quantify hip, knee, and ankle joint forces during exoskeletal-assisted walking (EAW) in the ReWalk P6.0, an FDA approved lower-extremity exoskeleton. It has 2 main objectives: The first objective was to quantify hip, knee, and ankle joint forces during unassisted walking and compare the results to existing in vivo and simulation data. The second objective was to compare hip, knee, and ankle joint forces from four different approaches to simulate human-robot interactions during EAW. OpenSim Moco is used to determine the joint reaction forces at the hips, knees, and ankles during unassisted walking and EAW. The study is well-developed and the results are intriguing. Once the following comments are addressed, the manuscript is recommended for publication.*

*Here are several technical review questions that need to be answered.*

1. *Page 6 line 133: What are the training protocols for the experiment? Describe here or at least provide it in supplementary document?*

Authors’ Response: We added a few sentences elaborating on the training protocol for this experiment in the “Exoskeletal-assisted walking training” section.

2. *Page 7 line 142: What is the experiment procedure, describe briefly?*

Authors’ Response: We added a few sentences under “Motion capture experiments during unassisted walking and EAW” section to elaborate on the experimental procedure and equipment used during the motion capture session. We clarified the participant’s tasks in the next paragraph of the same section. We also added Figure 2 to explain our experimental procedures.

3. *Page 8 line 180: What are the other cost terms here? Why you are using Opensim MOCO, as static optimization or CMC also have the same cost function of minimizing the muscle activation? Describe your cost function properly?*

Authors’ Response: We have clarified in the text that we used two default cost terms from OpenSim Moco’s *MocoInverse* tool: 1) minimizing the sum of squared muscle activations, and 2) constrain initial activations to initial excitations for each muscle. In addition, we modified the cost function to include a third cost term, which is EMG tracking of the eight muscles for which experimental EMG data were collected.

Next, we chose OpenSim Moco over static optimization or CMC due to its robustness in solving optimization problems (De Groote et al. 2016) and incorporating muscle dynamics (Hicks et al. 2015; Dembia et al. 2020; Denton and Umberger 2023). This is clarified in the text.

4. *Page 9 line 194: It is not clear how joint reaction force is quantified?*

Authors’ Response: We have clarified that joint reaction forces comprised the contributions of external forces and forces from all muscles crossing that joint.

5. *Page 10: What assumptions and parameter were made in Modeling the bushing forces and prescribed torques applied by the exoskeleton?*

Authors’ Response: We added several sentences to “Method 2: EAW (Prescribed Torques)” and “Method 3: EAW (Bushing Forces)” sections to clarify the assumptions and parameters.

6. *Page 10: How were interaction forces (in the bushing model) distributed across body segments, and how sensitive are the results to these assumptions?*

Authors’ Response: We have clarified in the text that the interaction forces were distributed across body segments consistent with the locations of the straps and pelvic band. These locations were obtained from experimental data. The distribution of bushing elements in the musculoskeletal model was as follows: one at the torso, two at each thigh, and one at each shank. Since we did not have to assume these locations, a sensitivity analysis was not needed.

7. *Page 12 line 250-255: It is redundant? Justify here.*

Authors’ Response: We purposefully added this redundancy for clarity. Here, we highlight the differing contributions from each simulation approach to the computed joint moments.

8. *Legend of figure should be clearer. As you explain in figure title at least mention all 3 lines in legend of figure 2.*

Authors’ Response: This figure is now Figure 3. We modified Figure 3 to add a legend for each sub-plot. We also modified the figure caption to include the references for each *in vivo* and simulation study.

9. *Page 22 line 482: Why joint force at hip and knee for assisted and unassisted is same?*

Authors’ Response: To clarify, the computed joint forces from one simulation approach, EAW (No Interactions), were similar to unassisted walking. This is because this simulation approach does not include the exoskeleton contributions. The EAW (No Interactions) approach required the same set of input parameters (joint kinematics, experimental EMG, and measured ground reaction forces) as unassisted walking. We added these sentences in the fourth paragraph of Discussion.

**Reviewer #4**

*This research article focuses on developing a computational framework to quantify the hip, knee, and ankle joint forces during exoskeleton-assisted walking. Although the manuscript is well-written, I have the following concerns, which may help the authors to improve the article.*

1. *The motivation behind this research is not clearly highlighted in the manuscript. There exist standard protocols as well as real-time approaches to evaluate joint torque during walking. This manuscript did not adequately justify how this work contributes in comparison to the existing literature that quantified joint moments.*

Authors’ Response: We have modified the Introduction and added several new references to strengthen the motivation. Next, we are unsure of the standard protocols the Reviewer is referring to in this comment. We have assumed that the Reviewer is referring to the use of inertial measurement units (IMUs) to estimate joint torques; we have not used IMUs in this study. We respectfully clarify that joint torques are not part of our research objectives. Our comparisons to prior work in this manuscript were limited to joint forces to be consistent with our research objectives.

2. *How different and novel is this framework compared to the existing studies that can evaluate the joint moments during assisted walking?*

Authors’ Response: We again point out that joint moments were not part of our research objectives. However, our framework to compute joint moments during EAW in an FDA-approved exoskeleton is novel. We are not aware of any other study that has computed joint moments in any FDA-approved exoskeleton.

3. *The framework used for custom-made exoskeletons can be utilized to measure the joint moments for other types of exoskeletons. What difference will the proposed framework make?*

Authors’ Response: Yes, it is plausible that a framework used for custom-made exoskeletons may be applicable to measure joint moments for other types of exoskeletons. We again point out that joint moments were not part of our research objectives. Our proposed framework provides a logical and rigorous approach to compute joint forces during EAW in an FDA-approved exoskeleton. FDA approval is important because patients only have access to FDA-approved devices in clinics and for in-home use. We have expended substantial effort to conduct controlled experiments and develop subject-specific virtual simulators of unassisted walking and EAW. These novel data allowed us to validate the virtual simulators with existing *in vivo* and simulation studies. As part of this manuscript publication, we will freely disseminate our dataset so the research community may build on our work to improve user experience and build better exoskeletons. No such dataset is currently available for EAW in any FDA-approved exoskeleton. We have explained our novel contributions throughout the manuscript.

4. *The study was validated with one subject, which raises questions about whether this framework works for a different subject or a variety of subjects.*

Authors’ Response: Yes, this framework will work for other able-bodied participants and participants with SCI. We have mentioned in our first limitation that we are actively working on expanding this computational framework to other participants. We have added a supplemental figure as pilot data from a participant with SCI during EAW.

5. *The methodology needs to be explained more clearly with figures of the experiment with the real subject.*

Authors’ Response: Per the Reviewer’s request, we have added another methods figure (Figure 2). We have included a silhouette of the participant during experiment and not a photo of the actual participant based on the guidelines of medRxiv.

6. *What conclusions can be drawn from Fig. 2? Is it only the results or a comparison with the earlier studies?*

Authors’ Response: This figure is now Figure 3. Figure 3 answered our first research objective, which was to quantify hip, knee, and ankle joint forces during unassisted walking and compare the results to existing *in vivo* and simulation data. In other words, Figure 3 does both – provides new results to address the first research objective and compares these new results to prior *in vivo* and simulation studies. We have clarified in the manuscript that this step was performed to validate and gain confidence in our computational framework in the absence of *in vivo* data during EAW.

**References**

De Groote, F., A. L. Kinney, et al. (2016). "Evaluation of Direct Collocation Optimal Control Problem Formulations for Solving the Muscle Redundancy Problem." Ann Biomed Eng **44**(10): 2922-2936.

Dembia, C. L., N. A. Bianco, et al. (2020). "OpenSim Moco: Musculoskeletal optimal control." PLoS Comput Biol **16**(12): e1008493.

Denton, A. N. and B. R. Umberger (2023). "Computational performance of musculoskeletal simulation in OpenSim Moco using parallel computing." Int J Numer Method Biomed Eng **39**(12): e3777.

Fournier, B. N., E. D. Lemaire, et al. (2018). "Modeling and Simulation of a Lower Extremity Powered Exoskeleton." IEEE Trans Neural Syst Rehabil Eng **26**(8): 1596-1603.

Haubert, L. L., D. D. Gutierrez, et al. (2006). "A comparison of shoulder joint forces during ambulation with crutches versus a walker in persons with incomplete spinal cord injury." Arch Phys Med Rehabil **87**(1): 63-70.

Hicks, J. L., T. K. Uchida, et al. (2015). "Is my model good enough? Best practices for verification and validation of musculoskeletal models and simulations of movement." J Biomech Eng **137**(2): 020905.

Perez-Rizo, E., F. Trincado-Alonso, et al. (2017). "Application of a model to analyze shoulder biomechanics in adult patients with spinal cord injury when walking with crutches in two different gait patterns." NeuroRehabilitation **40**(1): 129-140.

Segura, A. and S. J. Piazza (2007). "Mechanics of ambulation with standard and spring-loaded crutches." Arch Phys Med Rehabil **88**(9): 1159-63.

Smith, A. J. J., B. N. Fournier, et al. (2020). "Estimating upper extremity joint loads of persons with spinal cord injury walking with a lower extremity powered exoskeleton and forearm crutches." J Biomech **107**: 109835.
